# Açaí Seeds for a Greener Future: Transforming Agro-Waste into Industrial Value

**DOI:** 10.3390/foods15111967

**Published:** 2026-06-02

**Authors:** Karolynne Sousa Gomes, Maria Cecilia Pacco-Huamani, Michele Greque de Morais, Thaisa Duarte Santos, Jorge Alberto Vieira Costa

**Affiliations:** 1 Laboratory of Biochemical Engineering, College of Chemistry and Food Engineering, Federal University of Rio Grande, Rio Grande 96203-900, Brazil; 2 Laboratory of Microbiology and Biochemistry, College of Chemistry and Food Engineering, Federal University of Rio Grande, Rio Grande 96203-900, Brazil

**Keywords:** açaí seed, dietary fiber, green industry, bioeconomy, functional ingredients, eco-innovation

## Abstract

The growth in açaí consumption and exports has increased waste generation, particularly from the seed, which accounts for approximately 85% of the fruit mass and is frequently discarded improperly, causing adverse environmental impacts. In this context, the valorization of açaí seeds as a raw material represents a promising and environmentally sustainable alternative. Recent studies indicate that the chemical composition of açaí seeds, characterized by high fiber content and antioxidant compounds, underlies bioactive properties with potential applications across multiple industrial sectors. Therefore, this review aims to provide an overview of the composition and industrial applications of açaí seeds, while identifying current gaps and challenges. Available evidence suggests that incorporating açaí seed flour or extracts into food formulations is promising, although the observed effects are concentration-dependent. In addition, seed-derived extracts have demonstrated biological activities associated with potential health benefits. Furthermore, açaí seeds have potential applications as biochar for soil remediation and as adsorbents in water and wastewater treatment. However, the use of this by-product in packaging materials and in the energy sector still requires further investigation to achieve industrial-scale feasibility. Overall, the valorization of açaí seeds supports more sustainable industrial practices and aligns with circular economy principles.

## 1. Introduction

Açaí (*Euterpe oleracea* Mart.), an Amazonian fruit widely appreciated in Brazil and internationally, has seen increasing demand in recent years. The most recent data indicate an extractive production of 238,900 tons, with a total value of BRL 853.1 million (approximately USD 171 million) in 2023 [1]. However, when both extractive and cultivated sources are considered, total national production reaches approximately 1.94 million tons. Considering the morphological composition of the fruit, in which the pulp represents only 15% of the total fruit mass whereas the seed accounts for the remaining 85%, this production volume results in substantial waste generation, with an estimated 1.6 million tons of seeds discarded annually [2].

During pulp processing, these seeds are frequently discarded improperly in public areas, rivers, and landfills, causing pollution, habitat degradation, and potential damage to aquatic ecosystems [3,4]. In addition, the decomposition of açaí seeds may enrich water bodies with nutrients, promoting eutrophication and reducing dissolved oxygen levels, thereby harming fish and other aquatic organisms [5]. Growing awareness of climate change has encouraged the adoption of more sustainable industrial practices, increasing interest in converting this residue into value-added resources [1].

Several studies have demonstrated the application potential of açaí seeds. Their chemical composition is characterized by high fiber content, antioxidant compounds, and fatty acids, which are associated with relevant bioactive properties [3,6,7]. These characteristics support their potential use as functional ingredients [8,9], in the production of active packaging [10], and in health-related applications [11]. Açaí seeds have also been investigated for applications in the renewable energy sector [12] and in biochar production [13], particularly for sustainable agriculture and water and effluent treatment, in line with global innovation and sustainability goals [14].

Although several reviews have addressed açaí, none has focused exclusively on açaí seeds. Therefore, this review aims to provide an overview of the chemical composition and potential industrial applications of açaí seeds, while identifying current gaps and challenges. The review also highlights the potential contribution of açaí seed valorization to several Sustainable Development Goals (SDGs), particularly Goal 3 (Good Health and Well-Being), Goal 9 (Industry, Innovation and Infrastructure), Goal 11 (Sustainable Cities and Communities), Goal 12 (Responsible Consumption and Production), Goal 13 (Climate Action), and Goal 15 (Life on Land).

## 2. Methodology

**Figure 1:**
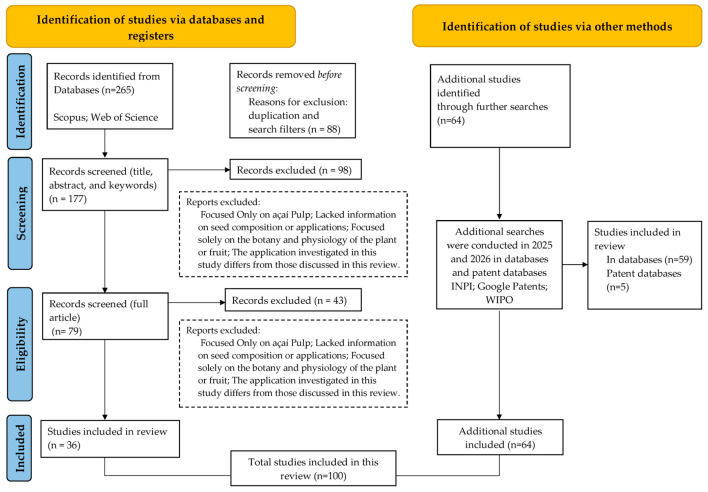
PRISMA Diagram of the Study Selection Process Used in the Development of This Review.

The studies included in this review were identified through searches conducted in the Scopus and Web of Science databases using the terms “açaí”, “seed”, “application”, “composition”, and “antioxidant capacity”. The search strategy retrieved 265 records. After applying filters for publication type (experimental studies and review articles), publication period (2020–2024), and language (English and Portuguese), and removing duplicate records, 177 studies remained for further assessment. The exclusion criteria applied throughout the selection process included: exclusive focus on açaí pulp; absence of information on seed composition or applications; studies restricted to the botany and physiology of the plant or fruit; and studies addressing applications not directly related to the objectives of this review. Based on these criteria, the titles, keywords, and abstracts of the 177 studies were screened, resulting in the exclusion of 98 studies. The remaining 79 studies were selected for full-text reading. After full-text assessment, 36 studies from the initial database search were included in the review. Additional searches conducted in 2025 and 2026, including database updates and consultation of patent sources such as the National Institute of Industrial Property (INPI), Google Patents, and World Intellectual Property Organization (WIPO), identified 64 additional studies. Altogether, 100 studies were included in this review. Details of the study screening process are presented in a PRISMA flow diagram (Figure 1).

## 3. Açaí Supply Chain: From Production to Residue Generation

Açaí is cultivated in several South American countries, with Brazil standing out as the world’s leading producer and exporter. Pará is the leading açaí-producing state in Brazil, accounting for up to 68.1% of the country’s total output. According to the most recent data from the Brazilian Institute of Geography and Statistics (IBGE), Brazil has shown gradual growth in açaí fruit production, increasing from 1.4 million tons in 2018 to 1.7 million tons in 2024. This increase was even more pronounced in terms of production value, which rose from just over BRL 3 million in 2019 to BRL 7 million in 2024 [15].

The expansion of açaí production is directly related to increasing demand. In recent years, this demand has been driven by the fruit’s widely studied antioxidant and nutraceutical properties, which have attracted interest in both domestic and international markets [16]. In Brazil, the main consumer markets for açaí are located in the states of Pará, Amapá, São Paulo, and Rio de Janeiro [17,18]. Internationally, the United States, Australia, Japan, the Netherlands, and the United Kingdom are among the leading importers of açaí pulp [19].

**Figure 2:**
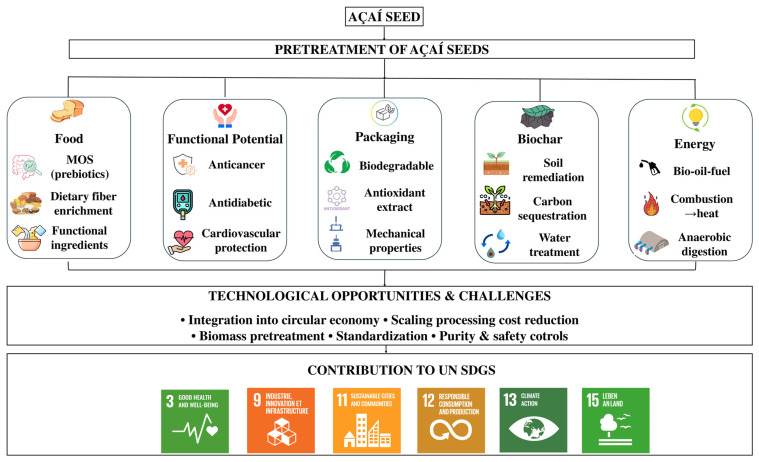
Outline of Applications for Açaí Seeds, Industrial Challenges, and Contribution to the SDGs.

The production of açaí pulp, one of the primary forms in which the fruit is commercialized and exported, involves several stages, including bunch harvesting, sanitization with a chlorinated solution, depulping, storage of the pulp in plastic containers for freezing, and transport for further processing. However, these stages generate various types of residues, such as inflorescences, peels, and seeds. Although sustainable alternatives have been reported for inflorescences, which can be converted into brooms [16], and for peels, which have potential as sources of antioxidant compounds [20], seeds are still largely discarded improperly, often on roadsides or in conventional waste streams, causing visual pollution and sanitary problems in urban environments [21]. Nevertheless, açaí seeds present several potential applications owing to their lignocellulosic composition and high fiber content, ranging from biochar production to the development of novel food products (Figure 2).

### Biochemical and Nutritional Characterization of Açaí Seeds

The composition of açaí seeds has been investigated to assess their potential for industrial applications. Table 1 summarizes the main results reported in the literature by different authors. The variability observed for some components may be attributed to differences in sample preparation, analytical methodologies, and the location and season of sample collection [3,22].

Differences in ash content observed in açaí seeds may be attributed to factors such as ripening stage, harvest period, and genetic variability among samples [25]. Most studies report ash contents below 2% in açaí seed biomass, which may favor energy applications and the production of value-added chemicals by reducing the need for additional pretreatment steps [26].

Furthermore, the presence of minerals such as potassium, magnesium, calcium, and iron indicates the potential of açaí seeds for use in food formulations with nutraceutical value, since the reported concentrations correspond to at least 15% of the recommended daily intake for adults and children according to the Brazilian Health Regulatory Agency (ANVISA) and the SDGs [32,33].

The seed is the fruit fraction with the highest fiber content, most of which is insoluble [23]. Insoluble fibers are recognized for promoting intestinal health, helping prevent digestive disorders, and contributing to overall well-being. Thus, the high fiber content of açaí seeds highlights the potential of this residue for industrial applications, particularly in supplementation and in the development of novel food products with health benefits [3,23,24].

The lignin content reported by Oliveira et al. [24] and Barros et al. [27] suggests that açaí seed biomass has potential for biochar and bio-oil production owing to its thermal resistance, an important characteristic for thermochemical processes such as pyrolysis [24].

The studies presented in Table 1, with the exception of Barros et al. [27], identified hemicellulose as the major component, differing from earlier findings in which cellulose predominated [34,35]. The cellulose and hemicellulose contents of açaí seeds also indicate their potential for obtaining value-added products, such as sugars produced by hydrolysis [26], energy [36], or substrates for biotechnological processes [25].

Açaí seeds also contain several antioxidant compounds, which are listed in Table 2. Proanthocyanidins, mainly composed of catechin units, are known for their antioxidant and anti-inflammatory properties. Their B1 and B2 forms show high bioaccessibility (72% and 87%, respectively), indicating potential applications in functional foods [37] and pharmaceuticals [38]. In addition, catechin and epicatechin have been associated with cardiovascular and neuroprotective effects and with the prevention of oxidative stress [37,39].

Rutin, ferulic acid, and hispidulin are other notable compounds identified in açaí seeds, with antioxidant, anti-inflammatory, and antimicrobial activities. These compounds act on inflammatory markers induced by UVB and UV radiation, demonstrating photoprotective properties and reinforcing their potential for cosmetic and dermatological applications [40].

High free radical-scavenging capacities have been observed in in vitro chemical assays, including DPPH (24.1–73.4%) and ABTS (33.0–83.5%). Although these preliminary tests do not directly reflect antioxidant behavior in complex matrices, they indicate relevant antioxidant reactivity of açaí seed extracts, suggesting that they are promising candidates for further exploration in the development of active packaging aimed at extending food shelf life [42]. In addition, ORAC values (4082.16 μmol Trolox equivalents/g) and TEAC values (3835.44 μmol Trolox equivalents/g) reported by Martins et al. [38] also indicate potential applications in animal feed formulations. Together, these findings highlight the broad applicability of açaí seeds in the food industry for both human and animal consumption, particularly in the development of functional foods and feeds with enhanced nutritional value.

Overall, the antioxidant compounds present in açaí seeds show promising potential for applications in food science, pharmaceuticals, and cosmetics. As demand for natural antioxidants continues to grow, further studies on açaí seeds remain important for understanding their biological effects and advancing their industrial applications. The composition data available for açaí seeds also highlight the wide range of possibilities for reusing this residue across different industrial sectors.

## 4. Açaí Seed Applications Across Industry: Opportunities and Challenges

### 4.1. Valorization of Açaí Seeds in the Food Industry: Dietary Fiber and Antioxidants

Açaí seeds (*Euterpe oleracea*) have been identified as a promising source of fibers, including mannans, mannooligosaccharides (MOS), and inulin. This composition positions açaí seeds as a valuable resource for the extraction of compounds of interest with various applications in the food industry [26,43].

Açaí seeds contain significant amounts of hemicellulose, which may reach up to 48% and is mainly composed of mannans (52.46%), which can be hydrolyzed to obtain MOS [7,44,45]. MOS are short-chain carbohydrates consisting of 3 to 10 mannose units and can be obtained through enzymatic or chemical hydrolysis from microbial and plant sources. They exhibit prebiotic, antioxidant, anti-stress, antidiabetic, and anti-inflammatory properties [46].

Murillo-Franco et al. [26] demonstrated that the hydrolysis of açaí seeds yielded MOS concentrations ranging from 8 to 10 g/L of hydrolysate, with mannobiose as the predominant product. In a complementary study, Murillo-Franco et al. [8] produced MOS from açaí seed hydrolysis using immobilized mannanase and obtained a maximum MOS concentration of 20 g/L without pretreatment.

In addition to MOS, açaí seeds are also a source of inulin, a soluble dietary fiber composed of linear chains of fructose units [45]. Inulin has been studied for its prebiotic effects, helping improve digestive health and increasing mineral absorption [47]. Different methods have been proposed to extract inulin from açaí seeds through the breakdown and solubilization of polysaccharide fractions [48]. For example, Magalhães et al. [45] extracted inulin from açaí seeds using water under optimized conditions of 94.1 °C and a solid-to-solvent ratio of 10 g/135.25 mL, obtaining a maximum yield of 166.38 μg/g of açaí seed flour.

In this context, pretreatment plays a crucial role in the valorization of açaí seeds, as it can reduce the resistance of the biomass to hydrolysis, improve the availability of structural polysaccharides, and enhance the recovery of value-added compounds [49]. Current studies on açaí seeds have focused mainly on acid hydrolysis and sequential acid–enzymatic strategies, largely because of the high mannan content of this biomass and the need to improve its conversion into mannose and mannooligosaccharides [50,51]. These approaches have shown promising results, but they still rely on processes that are not fully aligned with sustainable industrial routes. Therefore, the development of sustainable pretreatment strategies for açaí seeds remains an important gap for future research.

Although laboratory-scale studies have shown promising results, the economic feasibility of extracting fibers from açaí seeds still requires further investigation to determine whether this approach can be effectively applied at large scale. The specific costs associated with scale-up and process feasibility remain insufficiently addressed in the current literature [8,26]. Nevertheless, açaí seeds are already recognized as an abundant and economically attractive raw material, while costs related to fiber extraction and purification are expected to be primarily associated with processing steps such as drying, milling, repeated solid–liquid separation, solvent and reagent consumption, the use of specific enzymes, and purification and lyophilization operations [25,43,50]. Interest in these extraction routes remains high, particularly because MOS obtained from fiber-rich matrices may offer important physiological and functional benefits when incorporated into food products [26,52].

Studies such as that of St-Onge et al. [52], who incorporated 4 g of MOS into a beverage and evaluated its effects over a 12-week intervention period, demonstrated a significantly greater reduction in body weight in male participants compared with the placebo group, with weight loss reaching approximately 6% versus 2.3% in controls. In addition, MOS consumption was associated with reductions in overall body circumference as well as in subcutaneous and visceral adipose tissue. Similarly, Kumao and Fujii [53] reported that the daily intake of a lower dose, namely 1.0 g of MOS administered in a liquid coffee beverage for two weeks, was sufficient to reduce lipid absorption and significantly increase fecal fat excretion. Taken together, these findings suggest that the incorporation of MOS into food products may simultaneously improve intestinal function and enhance fecal fat excretion, thereby contributing to favorable changes in body composition.

Although studies specifically evaluating the application of açaí seed-derived MOS in food matrices are still lacking, the available evidence highlights the potential of MOS as food ingredients. In this context, açaí seeds emerge as a promising source of functional compounds for the development of prebiotic foods, dietary supplements, and other health-oriented food products.

The growing demand for sustainable and functional food systems has intensified interest in the valorization of novel ingredients, particularly agro-industrial by-products with nutritional and bioactive potential [54]. Within this scenario, açaí seeds have attracted increasing attention from the food industry due to their high dietary fiber content and antioxidant capacity. Recent studies have shown that açaí seed-derived ingredients can improve the nutritional profile and functional properties of food products [9,55]. The main food applications of açaí seeds and their associated advantages and limitations are summarized in Table 3.

Bordulis et al. [9] developed shakes targeted at the elderly population using formulations containing açaí seed and pulp flour. Açaí seed flour was incorporated at two concentrations, 5% and 6%. The study demonstrated that the inclusion of this ingredient increased the fiber content of the shakes to values up to four times higher than those of the control formulation. In the control, insoluble and soluble fiber contents were 3.2% and 0.7%, respectively, whereas in the formulation containing 6% seed flour these values reached 10.7% and 4.7%, respectively. In addition, antioxidant activity, assessed by DPPH and ABTS radical-scavenging assays, increased by 37.8% in the formulation containing 5% seed flour and by 172.9% in that containing 6% seed flour, compared with the control.

Consistent with these findings, other food applications have demonstrated the technological feasibility and nutritional relevance of açaí seed incorporation. For instance, the development of a cereal bar using roasted and ground açaí seeds resulted in a product containing 7.41% total dietary fiber, exceeding the values reported for the commercial counterparts evaluated [57]. Similarly, the incorporation of açaí seed flour into cookie formulations at levels of 20%, 50%, and 70% promoted a progressive increase in fiber content, with the higher-inclusion formulations being classified as a source of dietary fiber [58].

Despite these nutritional advantages, the incorporation of açaí seed flour may also induce relevant changes in the technological properties of food matrices, affecting both structure and visual appearance. In fresh pasta, for example, the addition of açaí seed flour increased hardness from 331.9 N in the control formulation to 805.7 N in samples containing 5% flour, accompanied by an increase in chewiness [27]. Marked color changes were also observed, with fresh pasta showing a reddish-purple hue and cookies formulated with higher flour concentrations exhibiting darker reddish tones. These physical and visual modifications may directly influence sensory perception and define practical limits for ingredient incorporation, since consumer acceptability tends to decrease as the proportion of açaí seed flour increases, particularly due to residual flavor and pronounced changes in texture and color [27,58]. Therefore, thermal processing strategies, strict control of incorporation levels, and palatability adjustments are essential to maximize the nutritional benefits of açaí seed-derived ingredients while maintaining product acceptability.

Another relevant application of açaí seeds in food development is the formulation of beverages with sensory characteristics similar to those of coffee, commercially known as “açaí coffee.” In the studies by Lima et al. [59] and Silva et al. [55], roasted and ground açaí seeds were infused in water at 100 °C, resulting in a beverage that resembled traditional coffee in appearance, taste, and aroma. Antioxidant properties were assessed using the DPPH, ABTS, and ORAC methods. Silva et al. [55] reported values of 88% for DPPH, 98% for ABTS, and 122% for ORAC, whereas Lima et al. [59] observed lower values, namely 66.29% for DPPH, 45.21% for ABTS, and 31.46 µmol TE/g for ORAC. In addition, Silva et al. [55] reported that in vitro digestion increased tannin and flavonoid concentrations, while antioxidant capacity, as determined by the ORAC assay, remained stable. Assays using Caco-2 intestinal cells exposed to the digested beverage demonstrated low cytotoxicity and a cytoprotective effect, suggesting a potential protective role against oxidative stress at the cellular level.

Overall, the incorporation of açaí seeds, either as flour or as whole roasted and ground material, appears to be a viable and promising approach. However, several challenges still need to be addressed, particularly the standardization of processing steps involved in flour production and derived products. Such standardization is essential to avoid or minimize discrepancies among results reported in beverage production studies, which may be associated with the roasting process. This step is often performed manually, without strict control of temperature and time, factors that play a decisive role in the formation of phenolic compounds and in shaping the antioxidant profile of the final product.

### 4.2. Health-Promoting Properties of Açaí Seeds: Bioactive Compounds and Therapeutic Potential

Açaí seeds have been investigated for their potential in the prevention and treatment of various diseases due to the presence of bioactive compounds [60,61,62,63,64]. Açaí seed extracts are rich in polyphenols, including flavonoids such as flavanols (catechins and epicatechins) and proanthocyanidins (condensed tannins), which are known for their ability to scavenge free radicals and reduce oxidative stress, a condition directly associated with several chronic diseases [65,66]. Table 4 summarizes studies that have investigated the effects of açaí seeds on various health conditions.

The studies presented in Table 4 suggest that açaí seed-derived products may have potential against different cancer types, including breast, colorectal, and cervical cancer. These effects have been associated with the high antioxidant activity of açaí seed extracts, attributed to their polyphenol content [70,71]. Oils and emulsions derived from açaí seeds showed phenolic acid concentrations up to 3.4 times higher than those found in pulp extract, which may contribute to the antitumor effects observed in these studies [11,39].

Antidiabetic effects have also been reported. According to Bem et al. [69], the high concentrations of catechin, epicatechin, and polymeric proanthocyanidins in açaí seeds were associated with reduced intestinal glucose absorption in rats. These compounds also exert antioxidant effects that may protect beta cells against hyperglycemia-induced toxicity, thereby contributing to improved insulin sensitivity.

Antihypertensive and cardioprotective effects have also been reported. For example, treatment with açaí seed extract significantly reduced oxidative damage in the aorta and adverse cardiac remodeling in obese rats, owing to the high polyphenol content of the extract [65]. Similarly, Silva et al. [60] demonstrated that administration of açaí seed extract prevented the development of hypertension during pregnancy, an effect that may be related to the presence of polyphenols such as flavan-3-ols, which can be absorbed and metabolized in the intestine and may exert beneficial health effects.

Moreover, positive effects in the treatment of anxiety [62], intestinal mucosal inflammation [63], and renal damage [68] have been reported and associated with the presence of phenolic and polyphenolic compounds in açaí seed extracts. Overall, the available evidence highlights the potential of açaí seeds for the development of natural products aimed at the prevention or management of various diseases.

Despite the promising therapeutic potential of açaí seeds reported in the literature, important challenges remain. The currently available evidence is based predominantly on preclinical studies, particularly in vitro assays and animal models [11,39,60]. Therefore, the positive effects observed in these studies may not be directly translatable to humans, since the findings indicate that biological effects in vivo may depend largely on polyphenol metabolites rather than on the parent compounds. In addition, there is still uncertainty regarding which metabolites are actually responsible for the observed effects and whether they are produced in the same proportion in humans. Another relevant aspect is that most of the beneficial responses reported have been attributed to the polyphenols present in the seeds; however, their bioactivity depends on the intestinal microbiota for the bioconversion of polymeric proanthocyanidins into biologically active metabolites. As this conversion is both species-specific and subject-specific, alterations in the gut microbiota may modify polyphenol biotransformation and contribute to interindividual variability in biological response. Added to this is the frequent use of genetically homogeneous animal models, which are unable to adequately represent the clinical and microbiological complexity of human populations [47,60,63,65,66,67,69,70,71].

Another important challenge is the lack of standardization in extraction methods, which hampers direct comparison among studies. Polyphenols may be sensitive to heat, light, extraction time, and solvent proportions, and therefore variations in processing conditions, such as boiling time and evaporation temperature, may directly affect the structural integrity and yield of these bioactive molecules. Consequently, when different studies report varying degrees of efficacy in animal models, it becomes difficult to determine whether these differences reflect true biological variation, such as the complex absorption and microbiota-dependent metabolism of polyphenols, or whether they result from differences in the baseline phytochemical profiles of the extracts caused by distinct extraction techniques [60,62,64,69].

### 4.3. Natural Fiber and Polymer Extraction from Açaí Seeds for Biodegradable Packaging

Concerns about contamination and chemical risks associated with conventional plastics have intensified the search for environmentally friendly alternative materials [72]. In this context, the use of agro-industrial residues for the development of sustainable packaging has gained increasing relevance in recent years. Recent studies, such as that of Marchetti et al. [73], have demonstrated the feasibility of food by-product valorization by using reground pasta as a substrate for polyhydroxyalkanoate (PHA) production, highlighting the potential to convert such residues into biodegradable polymers. Similarly, Hoque and Janaswamy [74] explored the use of banana peel fiber for the production of biodegradable films with promising mechanical properties and high biodegradability. In addition, Regmi et al. [75] and Regmi and Janaswamy [76] investigated the use of soyhull lignocellulose for the development of sustainable packaging films, in which UV-protective and antioxidant properties were also observed, indicating their potential to extend the postharvest shelf life of fruits such as raspberries. Taken together, these findings highlight the growing relevance of agro-industrial waste valorization as a strategy for the development of sustainable packaging materials, in which açaí seeds also stand out as a promising lignocellulosic resource.

The composition of açaí seeds makes them a promising raw material for the development of biodegradable films [44]. Several studies [10,42,66] have shown the compatibility of açaí seeds with other natural polymers, allowing the formation of films for food applications. In addition, seed extracts have been used to improve the rheological properties of film-forming solutions and to increase their antioxidant capacity [10,42]. However, the studies mentioned in this review do not provide direct comparisons between films containing açaí seed flour or extract and conventional or bio-based packaging [42,66,77]. Unlike conventional synthetic packaging, whose mechanical strength, barrier performance, and industrial scalability are already well established, the currently available studies on açaí seed-based films are still limited and do not support a robust performance comparison [75,76]. Similarly, comparisons with other bio-based packaging matrices are also challenging, as the reported studies differ in terms of formulation, processing conditions, and parameters evaluated [66,77,78]. Therefore, while these materials are promising, more standardized studies are needed to determine their competitiveness relative to conventional packaging and other bio-based alternatives.

Romani et al. [66] demonstrated that incorporating açaí seed flour into broken rice starch films conferred antioxidant properties. Antioxidant activity increased with higher flour concentrations in the film composition, resulting in 12%, 30%, and 40% ABTS radical reduction for flour concentrations of 5%, 10%, and 15%, respectively. In this context, Bertolo et al. [10] evaluated the influence of adding açaí seed flour extract to starch- and gelatin-based film-forming solutions. The total phenolic content of the seed was 79 mg GAE/g, and these compounds positively influenced the rheological properties of the solutions. Viscoelastic stability increased from 36.33% in the control to 133.47% in the solution containing extract. In addition, the sample containing extract showed lower stiffness (14.33 Pa) than the control (42.24 Pa).

Nogueira et al. [42] investigated the development of films from red bean flour and açaí seed extract. The addition of 10% extract reduced film solubility from 37.6% in the control to 32.3% in the extract-containing formulation. Mechanical properties such as tensile strength and elongation also improved, reaching 4.7 MPa and 37.1%, compared with 2.3 MPa and 30.4% in the control, respectively. In addition, when applied as olive oil packaging, the films maintained quality parameters within Codex Alimentarius standards at 60 °C for 16 days, owing to their enhanced antioxidant activity. Similarly, Silva et al. [77] found that films developed with fibers extracted from açaí seeds showed resistance to penetration by oily mixtures and improved mechanical properties, with tensile strength reaching 97.2 MPa. However, these films also exhibited high water vapor permeability (494 g/day/m^2^) and may therefore be considered a promising alternative for packaging low-moisture products such as grains, snacks, and biscuits.

Braga et al. [78] evaluated nanostructured films produced with cellulose obtained from fibers of the açaí seed mesocarp. The results indicated that fibers subjected to three milling cycles yielded readily dissolvable films, suitable for instant foods. In contrast, fibers processed through 21 milling cycles produced water-resistant films suitable for use as secondary coatings for paper and cardboard. Furthermore, chemical treatments applied to the fibers increased the crystallinity index and removed non-lignocellulosic components, thereby improving mechanical and barrier performance. Overall, these findings indicate that açaí seeds, whether in the form of flour or extract, have considerable potential for the production of environmentally sustainable packaging with improved antioxidant and technological properties, supporting their application across various food systems. However, the main challenges involve moisture barrier properties, the need for formulation optimization, and the dependence on physical and chemical treatments, such as multiple milling cycles and procedures aimed at increasing crystallinity, removing non-lignocellulosic components, and improving mechanical and barrier performance. These factors may also pose challenges for scale-up, highlighting the need for greater standardization of production parameters to support consistent industrial application.

### 4.4. Biochar from Açaí Seeds: Valorization of Agro-Waste for Soil Improvement and Pollution Control

Biochar is a carbon-rich material produced through pyrolysis from woody biomass, agricultural and agro-industrial residues, manure, and leaves. This process involves heating organic biomass under limited oxygen conditions at temperatures below 900 °C [79,80]. In this context, açaí seeds, as an agro-industrial residue, have been investigated and have shown considerable potential for biochar production and application across a wide range of sectors, from agriculture to environmental management [81].

Studies on biochar produced from açaí seeds have revealed complementary aspects related to its production and properties. For example, Guerreiro et al. [82] found that pyrolysis temperatures between 400 °C and 450 °C considerably altered the structural organization of açaí seeds by breaking down their cellular structure, which is composed of cellulose, lignin, and hemicellulose. Such changes are essential for improving the physicochemical properties of the resulting biochar. Sato et al. [83] reported that, for soil applications, the optimal temperature for producing biochar from açaí seeds is 600 °C, with a residence time of approximately 60 min. Under these conditions, the resulting biochar showed increased recalcitrance, pH, and water retention capacity, all of which are important characteristics for use as a soil conditioner.

Applications of açaí seed biochar in agriculture, particularly in soil remediation, are among the most extensively studied. Biochar promotes carbon sequestration in the soil, thereby reducing greenhouse gas emissions from agriculture and contributing to climate change mitigation [79].

Mendonça et al. [84] evaluated the use of açaí seed biochar as a soil amendment and its effects on the development of black pepper seedlings. The results showed that biochar positively altered the physicochemical and biochemical properties of the soil, increasing its water-holding capacity and moisture retention, which are important parameters for plant growth. Biochar also enhanced soil enzymatic activity, increasing microbial activity and nutrient availability to the roots, which promoted significant growth of black pepper seedlings, particularly in terms of root development and plant height.

The mitigating effect of açaí seed biochar was also evaluated in soybean cultivation under drought conditions. The application of biochar at a concentration of 10% (*w*/*w*) yielded the best results for the physiological performance of soybean plants, resulting in a 121% increase in photosynthetic rate and an 88% increase in water-use efficiency. In addition, photosynthetic pigment levels increased, as did biomass production, leaf dry matter, and root dry matter, highlighting its important role in supporting plant health under water stress conditions [85].

In addition to agricultural applications, açaí seed biochar has shown potential in contaminant adsorption processes. Ramirez et al. [81] demonstrated the efficiency of this biochar in removing the herbicide atrazine from aqueous solutions. Removal efficiency reached 95% at pH 6.5 and an adsorbent concentration of 0.54 g/L. Similarly, Zaparoli et al. [13] reported glyphosate removal under acidic conditions, with an efficiency of up to 87.9%, using biochar impregnated with FeCl_2_ and FeCl_3_. These studies highlight the efficiency of açaí seed biochar against various contaminants under different environmental conditions, positioning it as a promising alternative for industrial effluent treatment.

However, although the studies presented identify açaí seeds as an abundant and low-cost residual biomass, these attributes alone do not guarantee the economic viability of thermochemical routes [83]. The availability of this feedstock is directly related to the fact that residues from fruit processing can account for up to 85% of its mass, reinforcing its potential as a raw material for pyrolysis and gasification processes [85]. In addition, the available literature reports values of approximately US$ 5.5 t^−1^ for this biomass, highlighting its attractive cost. Nevertheless, economic feasibility depends not only on the cost of obtaining the biomass, but also on the technological route adopted, process yields, and scale of operation [86]. In this regard, a techno-economic assessment of the thermocatalytic pyrolysis of açaí seeds reported economic infeasibility under the investigated conditions, since the costs of biomass pretreatment, particularly chemical impregnation, combined with the low yield of the target product, resulted in operational losses [4]. Therefore, future studies on the pyrolysis and gasification of açaí seeds should integrate not only technical performance indicators but also economic indicators to more realistically define their industrial application potential.

### 4.5. Sustainable Energy from Açaí Residues: A Circular Approach to Waste to Power

Açaí seeds have attracted increasing attention as a renewable energy source due to their high lignin and cellulose contents, which are important components for thermochemical conversion processes such as pyrolysis and combustion [85,87,88]. In addition, the calorific value of açaí seeds is notable, reaching 21.1 MJ/kg, which is comparable to that of raw materials such as oak, hardwood, and pine, making them a viable option for energy generation and biofuel production [12,22].

The thermodynamic parameters evaluated during the pyrolysis of açaí seed biomass, including free energy and entropy, support its potential for energy generation, with average free energy values ranging from 148.76 to 148.81 kJ/mol [12]. Furthermore, pyrolysis of açaí seeds can yield bio-oil with a high hydrocarbon content, further supporting its viability as an energy source [89].

In addition to pyrolysis, other methods, such as anaerobic digestion, have also been explored for energy recovery from açaí seeds. In the study by Ampese et al. [90], direct anaerobic digestion of açaí seeds was investigated as a route for energy recovery. The results indicated a potential output of 3.4 MW of electrical power and 7.0 MW of thermal power, demonstrating the feasibility of energy recovery from this residue. Furthermore, the use of electricity derived from açaí by-products may reduce greenhouse gas emissions by approximately 3.7 t CO_2_-eq per year. This reduction is associated with the replacement of conventional energy sources by more sustainable alternatives, thereby contributing to climate change mitigation [90].

Although multiple studies have highlighted the potential of açaí seeds for renewable energy production, several challenges still limit their application for this purpose. Among these, the presence of polysaccharides such as mannan stands out, as it hinders the efficient hydrolysis of structural carbohydrates and requires the use of specific enzymes or acid-based treatments [51,91]. In this context, pretreatment approaches reported for the valorization of açaí seeds include dilute-acid hydrolysis [25,90], sequential acid–enzymatic treatments [90], alkaline delignification [34], and chemical strategies to improve the conversion of recalcitrant mannan into fermentable sugars [48]. However, pretreatment techniques increase process complexity and the overall energy production cost [36,92]. Therefore, further studies are needed to optimize pretreatment methods for açaí seed biomass conversion into energy.

## 5. Açaí Seeds and the Sustainable Development Goals

**Figure 3:**
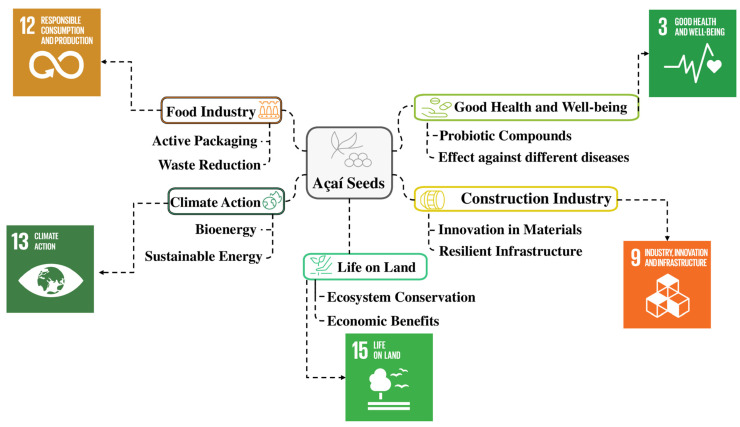
Schematic Representation of How the Industrial Applications of Açaí Seeds Relate to the Sustainable Development Goals.

The incorporation of açaí seeds into various industrial processes not only enhances economic valorization but also promotes environmental sustainability by reducing waste accumulation and encouraging residue reuse [66,93]. In addition, the use of açaí seeds aligns with several United Nations Sustainable Development Goals (SDGs), as illustrated in Figure 3.

In this context, the integration of açaí seeds into sustainable practices can make an important contribution to achieving these goals. Innovative approaches to the responsible and sustainable use of this residue are closely aligned with SDG 12 (Responsible Consumption and Production) and SDG 11 (Sustainable Cities and Communities). One example is the study by Romani et al. [66], in which açaí seed flour was used in the development of active food packaging, thereby reducing the improper disposal of seeds in the environment and adding economic value to this residue.

The development of technologies to obtain value-added products from açaí seeds, such as mannose and proanthocyanidins, illustrates innovative potential in the food industry. Studies such as those by Monteiro et al. [51] and Murillo-Franco et al. [8] have highlighted the potential of these seeds as a cost-effective source of compounds with prebiotic activity and high industrial demand. In addition, studies have shown the benefits of açaí seed extracts in the prevention and treatment of various diseases, particularly malignant neoplasms, suggesting the potential of açaí seed-derived products as functional ingredients [11,94]. These applications align with SDG 12 (Responsible Consumption and Production) and SDG 3 (Good Health and Well-Being).

Advances related to the use of açaí seeds and their extracts also extend to the construction sector, in line with SDG 9 (Industry, Innovation and Infrastructure). In the study by Martins et al. [95], açaí seed extract showed anticorrosive potential, highlighting its possible application in materials science and industrial innovation. This property may contribute to the development of more resilient infrastructure and foster technological innovation in the sector.

In the context of SDG 13 (Climate Action) and SDG 7 (Affordable and Clean Energy), the use of açaí seeds for bioenergy production offers a viable strategy for climate change mitigation, reducing environmental impacts, increasing the share of renewable energy sources in the global energy mix, and promoting sustainable energy practices in the Amazon region [90]. Moreover, the use of açaí seeds in industrial processes, together with the promotion of agroforestry practices, can contribute to the conservation of forest ecosystems while simultaneously generating economic benefits for local communities, in line with SDG 15 (Life on Land) [21].

Overall, the inclusion of açaí seeds in sustainable development strategies contributes to multiple SDGs. These contributions also highlight the importance of innovative approaches to residue management and efficient resource use, thereby supporting a more sustainable future.

## 6. Future Perspectives and Final Considerations

This review has presented and discussed various applications of açaí seeds. The growing demand for sustainable industrial solutions has increased interest in the valorization of this residue, suggesting strong future potential. However, several challenges still need to be addressed before current and future applications of açaí seeds can become feasible at a large scale.

In this context, studies conducted in the coming years are expected to focus on the development of environmentally sustainable pretreatments to enhance the industrial applications of this raw material in the energy sector and improve the extraction of valuable chemical compounds.

In the medical and pharmaceutical fields, the prospects are promising. An increase is expected in the number of studies focused on the development of novel herbal medicines based on açaí seed extracts. Furthermore, research aimed at elucidating the mechanisms of action of these extracts against various diseases, particularly cancer, is also expected to expand.

The production of packaging incorporating açaí seed flour, rather than only its extract, still faces challenges and requires further investigation. Among the aspects that need to be addressed are the concentration of flour incorporated into film-forming solutions, particle size, film production conditions, film stability and uniformity, storage stability, and the feasibility of industrial application.

In the food sector, the possibilities are also highly promising. An increase is expected in the number of studies aimed at developing novel foods or incorporating açaí seed flour into formulations designed to improve nutritional composition, especially owing to its high fiber content. For this reason, new patents are expected to emerge in the coming years, particularly those related to the use of açaí seeds in innovative food products, considering the technological potential of the fibers and bioactive compounds present in this raw material.

Current patents are mainly focused on extract production techniques and health-related applications aimed at the development of cosmetics and pharmaceuticals [96,97]. Some patents also report methods for extracting oils from açaí seeds and their applications [98,99]. In addition, patents related to the development of food products using açaí seeds, including beverages and coffee-like products [98,100], have gradually increased.

Overall, the available evidence highlights the broad potential of açaí seeds for diverse applications, with substantial opportunities for future research and for the development of strategies that promote their efficient and sustainable use.

## Figures and Tables

**Table 1:** Proximate Composition on a Dry Basis and Mineral Profile of Açaí Seeds.

Ash	Lipids	Soluble Fiber	Insoluble Fiber	Cellulose	Hemicellulose	Lignin	Reference
2.09%	1.75%	Not reported	Not reported	12.65%	42.58%	11.41%	[6]
1.36%	2.75%	0.99%	85.21%	Not reported	Not reported	Not reported	[3]
7.54%	2.98%	Not reported	Not reported	13.05%	42.67%	15.91%	[22]
1.52%	2.03%	2.24%	53.77%	Not reported	Not reported	Not reported	[23]
1.44%	Not reported	Not reported	Not reported	Not reported	51.94%	34.04%	[24]
1.52%	1.9%	Not reported	Not reported	3.6%	58%	11.6%	[25]
1.57%	2.64%	Not reported	Not reported	11.58%	37.88%	14.12%	[26]
0.96%	3.5%	Not reported	Not reported	8.5%	48.1%	16.4%	[7]
1.01%	Not reported	Not reported	Not reported	45.49%	21.08%	24.36%	[27]
2.08%	2.38%	Not reported	Not reported	12.01%	39.03%	12.99%	[28]
Minerals							
Sodium	Potassium	Magnesium	Phosphorus	Calcium	Iron	Manganese	
3.16%	47.95%	8.75%	13.81%	19.50%	Not reported	Not reported	[29]
17 mg/100 g	273.3 mg/100 g	56.7 mg/100 g	16.22 mg/100 g	281.9 mg/100 g	1.5 mg/100 g	2.83 mg/100 g	[30]
6.06 mg/100 g	685.9 mg/100 g	86.13 mg/100 g	Not reported	131.6 mg/100 g	30.37 mg/100 g	58.73 mg/100 g	[31]

**Table 2:** Antioxidant Compounds in Açaí Seeds.

Antioxidant Compound	Origin	Concentration	Suggested Applications	Reference
Procyanidins B1	Açaí seeds	16.08 mg/g	Bioactive compounds for the development of new functional foods.	[37]
Procyanidins B2		1.49 mg/g		
Catechin		15.67 mg/g		
Epicatechin		5.32 mg/g		
Procyanidins B1	Açaí seeds	16.08 mg/g	Natural antioxidant for emulsions and meat products.	[3]
Procyanidins B2		1.49 mg/g		
Catechin		15.66 mg/g		
Rutin	Açaí seeds	69.86 µg/g	Antioxidant cosmetics with anti-aging, photoprotective, soothing, and anti-sagging properties.	[40]
Ferulic acid		41.6 µg/g		
Hispidulin		48.61 µg/g		
Polyphenols	Açaí seeds	26.60 μMol EQ/g	Therapeutic use in traditional medicine for chronic inflammatory conditions.	[41]
Catechin	Açaí seeds	Not reported	Pharmaceutical and cosmetic industries, or as an additive for animal feed products.	[38]
Epicatechin				
Total phenols	Nanoemulsion	146.00 mg EAG/g	Cardiovascular disease, vascular inflammation.	[39]
Total flavonoids		113.80 mg EQ/g		

**Table 3:** Food Applications of Açaí Seeds and the Main Reported Advantages and Limitations.

Application Form	Seed Concentration	Food Product	Main Advantages	Main Limitations	Reference
Flour	5% and 6%	Shakes	Showed high insoluble fiber content, contributing to gastrointestinal health. Increased antioxidant activity.	Were not reported.	[9]
Roasted and ground seed	10% (*w/v*)	Caffeine-free coffee-like beverage	Contained phenolic compounds and showed antioxidant activity in DPPH (88%), ABTS (98%), and ORAC (122%) assays, in addition to cytoprotective potential.	Showed a 23% reduction in DPPH activity and a 54% reduction in ABTS activity after in vitro digestion.	[55]
Flour	5% to 10%	Fresh tagliatelle-type pasta	Did not increase cooking time.	Reduced water absorption and increased hardness and chewiness.	[56]
Roasted and ground seed	Not reported	Cereal bar	Increased fiber and mineral contents. Reduced caloric value compared with the control.	Required labor-intensive pretreatment.	[57]
Flour	20%, 50% and 70%	Cookies	Increased fiber content, meeting the criteria for classification as a source of fiber.	Negatively affected color, sensory acceptance, and purchase intention at high incorporation levels.	[58]
Aqueous extract of roasted seeds	6% (*w*/*v*)	Beverages	Showed the presence of bioactive compounds, including phenolics, amino acids, and organic acids.	Showed a loss of total phenolic compounds after the intestinal phase of digestion.	[59]

**Table 4:** Açaí Seeds and Their Reported Health Benefits: Study Models and Applied Doses.

Sample	Studied Aspect	Study Model	Animal Model	Applied Dose	Health Benefits	Reference
Lyophilized extract	Antioxidant; anti-inflammatory	In vivo	Male C57BL/6 mice	300 mg/kg/day	Reduced cardiovascular oxidative damage and hyperglycemia.	[65]
Extract	Antitumor (breast cancer)	In vivo	Wistar rats	100, 300, and 1000 mg/kg	Reduced tumor size compared with the negative control group; decreased the proliferative index and promoted 95% tumor necrosis.	[67]
Oil	Antitumor (colorectal adenocarcinoma)	In vitro	Not applicable	100 μg/mL	Reduced the viability of Caco-2 and HCT-116 cancer cells and increased apoptosis after 24 h of treatment.	[11]
Oil and nanoemulsion	Anticancer (cervical cancer) Cytotoxicity	In vivo and in vitro	Female Swiss mice	50 μg/mL of oil	Reduced the number of viable cells by 62.1% in HeLa cells and by 68.8% in SiHa cells compared with the negative control.	[39]
Lyophilized extract	Renoprotective and antifibrotic	In vivo	Male CD1 Swiss mice	350 mg/kg/day	Reduced markers associated with renal fibrosis and improved oxidative damage, fibrosis, and renal function.	[68]
Lyophilized extract	Improved exercise performance in aging	In vivo	Male Wistar rats	200 mg/kg/day	Increased running time by 73% compared with the initial value, restored aortic damage, and enhanced antioxidant defense.	[61]
Lyophilized extract	Anxiolytic	In vivo	Wistar rats	200 mg/kg/day	Exerted anxiolytic effects, improved neurogenesis, and reduced stress hormones and oxidative damage in the brainstem.	[62]
Lyophilized extract	Antidiabetic	In vivo	Male Wistar rats	200 mg/kg/day	Increased insulin receptor expression in adipose tissue and corrected metabolic alterations such as elevated blood glucose, serum insulin, and HbA1c levels.	[69]
Lyophilized extract	Effect against non-alcoholic fatty liver disease	In vivo	Male C57BL/6 mice	300 mg/kg/day	Reduced oxidative damage in liver tissue and hyperglycemia, reaching levels similar to those of the control group.	[64]
Lyophilized extract	Effect on preeclampsia symptoms	In vivo	Female Wistar rats	200 mg/kg/day	Prevented hypertension, endothelial dysfunction, and microalbuminuria, and reduced placental and fetal mass.	[60]
Lyophilized extract	Effect on the inflammatory and functional aspects of intestinal mucositis	In vivo	Male Swiss mice	100 mg/kg	Attenuated the inflammatory response associated with intestinal mucositis, protected the intestinal mucosal barrier, and reduced inflammation.	[63]

## Data Availability

No new data were created or analyzed in this study. Data sharing is not applicable to this article.
